# Serum angiopoietin is associated with lung function in patients with asthma: a retrospective cohort study

**DOI:** 10.1186/1471-2466-14-143

**Published:** 2014-09-02

**Authors:** Kuk-Young Moon, PuReun-HaNeul Lee, Sung-Woo Park, Choon-Sik Park, An-Soo Jang

**Affiliations:** 1 Genome Research Center for Allergy and Respiratory Diseases, Soonchunhyang University Bucheon Hospital, 170 Jomaru-ro, Wonmi-gu, Bucheon, Gyeonggi-do 420-767, Republic of Korea; 2 Department of Internal Medicine, Division of Allergy and Respiratory Medicine, Soonchunhyang University Bucheon Hospital, 170 Jomaru-ro, Wonmi-gu, Bucheon, Gyeonggi-Do 420-767, South Korea

**Keywords:** Angiogenesis, Angiopoietin-1, Angiopoietin-2, Asthma

## Abstract

**Background:**

Angiopoietin-1 (Ang-1) is an essential mediator of angiogenesis that establishes vascular integrity, and angiopoietin-2 (Ang-2) acts as its natural inhibitor. We considered that angiopoietin might be important in bronchial asthma.

**Methods:**

In total, 35 patients with asthma and 20 healthy subjects were studied.

**Results:**

The serum Ang-1 levels were significantly elevated in patients with asthma compared to control subjects (293.9 ± 13.8 pg/mL vs. 248.3 ± 16.2 pg/mL, respectively, p = 0.04). The serum Ang-2 levels were not different between the two groups. The areas under the curve (AUC) for serum angiopoietins revealed that the serum level of Ang-1 (0.68) was more sensitive and specific than the serum Ang-2 level (0.55) for differentiating between patients with asthma and control subjects. The serum Ang-1/Ang-2 ratio was correlated with the FEV1/FVC ratio (r = -0.312, p = 0.02), while serum Ang-2 was correlated with body mass index.

**Conclusions:**

Our results indicate that the serum Ang-1 levels were higher in asthma patients compared with healthy subjects. As the Ang-1/Ang-2 ratio was related to lung function, the data suggest that serum angiopoietin is associated with lung function in patients with asthma.

## Background

Increased angiogenesis is a well-documented feature of airway remodeling in asthma [1-6]. Angiogenesis is defined as the formation of new blood vessels from pre-existing endothelium. Airway remodeling [2] in asthmatic patients involves a wide array of pathophysiological features, including epithelial changes, increased smooth muscle mass, increased numbers of activated fibroblasts/myofibroblasts, subepithelial fibrosis, angiogenesis, alterations in extracellular matrix components, and vascular changes. Multiple cytokines, chemokines, and growth factors released from inflammatory and structural cells in airway tissue create a complex signaling environment that drives these structural changes. Previous studies suggested that neovascularization and microvascular leakage are prominent in asthmatic airways [2-7]. Vascular endothelial growth factor (VEGF) is one of the most potent proangiogenic factors [8]. Angiogenesis can be initiated by endogenous angiogenic factors released from mesenchymal cells and/or inflammatory cells [9]. Under physiological conditions, angiogenesis is controlled by the equilibrium between the proendogenous and antiendogenous angiogenic factors released from the extracellular matrix [10]. In experimental models, administration of angiopoietin-2 (Ang-2) provokes microvascular leakage in the lungs and other organs [11,12], whereas Ang-1 protects against microvascular leakage induced by VEGF, Ang-2, or inflammatory agents [13-16]. Ang-2 is released from endothelial cells [17] and human monocytes [18], while various other tissue cells produce Ang-1. Both Ang-1 and Ang-2 bind to Tie2 receptor, which is abundantly expressed in lung endothelium [19]. It was reported that Ang-1 and Ang-2 levels were increased in sputum supernatant in severe refractory asthmatics and mice [20-22]. Therefore, we examined the serum levels of angiopoietins in patients with asthma and investigated the possible associations with physiological variables.

## Methods

### Subjects

We retrospectively studied clinical data on 35 asthma patients (Table 1) who were registered in an asthma cohort of the Genome Research Center for Allergy and Respiratory Diseases in Korea. All patients were recruited from Soonchunhyang University, Bucheon Hospital. Asthma diagnoses were based on GINA guidelines [23]. All subjects had a clinical diagnosis of asthma that was supported by one or more of the following criteria: 1) variability in the maximum diurnal peak expiratory flow of greater than 20% over the course of 14 days, 2) an increase in FEV1 of greater than 15% after the inhalation of 200-400 μg of albuterol, or 3) a 20% reduction in FEV1 in response to a provocative concentration of inhaled methacholine (PC20 methacholine) of less than 10 mg/mL. All subjects underwent standardized assessments, which included analyses of induced-sputum specimens, a complete blood cell count with a differential count, IgE measurement, chest posteroanterior radiography, allergy skin prick tests, and spirometry. All data were collected at the time of diagnosis, before the administration of asthma medication.

**Table 1:** Clinical and physiological variables in patients with bronchial asthma and control subjects

	**Normal controls**	**Asthmatic patients**
**Variables**	**n = 20**	**n = 35**
Age, yr	51.4 (±8.9)	46.6 (±13.7)
Sex (M/F)	13/7	15/20
Smoke (SM/ES/NS)	5/2/10	5/8/20
Atopy (Y/N)	6/14	17/18
Asthma period, yr	0	1.53 (±0.72)
IgE (pg/ml)	72.08 (±84.0)	260.11 (±514.0)
BMI (kg/m^2^)	23.72 (±2.89)	23.97 (±3.01)
FVC (%, predicted)	91.40 (±9.1)	73.00 (±14.6)*
FEV_1_ (%, predicted)	103.0 (±8.7)	74.0 (±16.6)*
FEV_1_/FVC %	86.0 (±4.9)	74.9 (±10.8)*
PC20 methacholine	23.87 (±5.05)	3.11 (±4.63)*

*p < 0.001 compared with control subjects.

Plus-minus values are mean ± S.D. PC20 methacholine; the concentration of methacholine required to decrease the FEV1 by 20%, FEV1; forced expiratory volume in one second. FVC; forced vital capacity. BMI; body mass index. SM; smoker, ES; ex-smoker, NS; non-smoker.

Asthmatic subjects matched to normal controls in terms of age, sex, and body mass index (BMI) were selected from among the asthma cohort of Bucheon Hospital for inclusion in this study. The normal control subjects were spouses of the patients or members of the general population who answered negatively to a screening questionnaire regarding respiratory symptoms and other allergic diseases, had a predicted FEV1 value greater than 80%, a PC20 methacholine greater than 10 mg/mL, and normal findings on simple chest radiographs. The exclusion criteria included respiratory infection during sputum induction, chronic obstructive pulmonary disease (COPD), vocal cord dysfunction, obstructive sleep apnea, Churg-Strauss syndrome, cardiac dysfunction, allergic bronchopulmonary aspergillosis, and poor adherence to treatment. The local research ethics committee of the Soonchunhyang University Hospital Research Board approved the study protocol. All patients and control subjects were recruited over a period of 3 months. Serum samples were collected at the time of diagnosis before the administration of asthma medication and at the time of control subject recruitment to minimize bias due to the storage time of the patient samples and the measurement of serum angiopoietins.

### Lung function analyses

Spirometry [24] was performed before and after bronchodilator use. Baseline FVC and FEV1 measurements were obtained in the absence of bronchodilator use (within 8 h). Basal and post-bronchodilator FEV1 and FVC were measured. We used a Vmax Series 2130 Autobox Spirometer (Sensor Medics Corp., Yorba Linda, CA, USA) and performed a calibration check every morning at 8 am.

### Allergy skin tests

Skin prick tests were performed using 24 common inhalant allergens, including dust mites (*Dermatophagoides farinae* and *Dermatophagoides pteronyssinus*), cat fur, dog fur, cockroaches, grass, tree, pollens, ragweed, and *Aspergillus* species (Bencard Co.; Brentford, UK) [25]. Atopy was defined as either having a wheal reaction from the allergen equal to or greater than the histamine wheal (1 mg/mL) or at least 3 mm in diameter. Total IgE was measured using the UniCAP system (Pharmacia Diagnostics, Uppsala, Sweden).

### BMI

The BMI of the subjects [26-28] was calculated as weight (kg)/height (m^2^).

### Mediator assays

Ang-1 and Ang-2 levels were assessed in serum supernatant samples using ELISAs according to the manufacturers’ instructions. The minimum detectable dose of Ang-1 and Ang-2 ranged from 1.36-10.3 pg/mL and 1.20-21.3 pg/mL (R&D Systems, Minneapolis, MN, USA), respectively. Blood was drawn to evaluate serum albumin levels using laser nephelometry. All values were expressed in pg/mL. For Ang-1 and Ang-2, the intra- and inter-assay variability values were 3.5% and 6.3%, respectively.

### Statistical analyses

The data were double entered into a statistical software package (SPSS, version 14.0; SPSS Inc., Chicago, IL, USA). The data are expressed as means ± standard deviation (SD). Group differences between the asthmatic patients and control subjects were compared using two-sample *t*-tests, Mann-Whitney tests, or Pearson’s chi-square tests for normally distributed, skewed, or categorical data, respectively. A value of p <0.05 was taken to indicate statistical significance.

## Results

**Figure 1:**
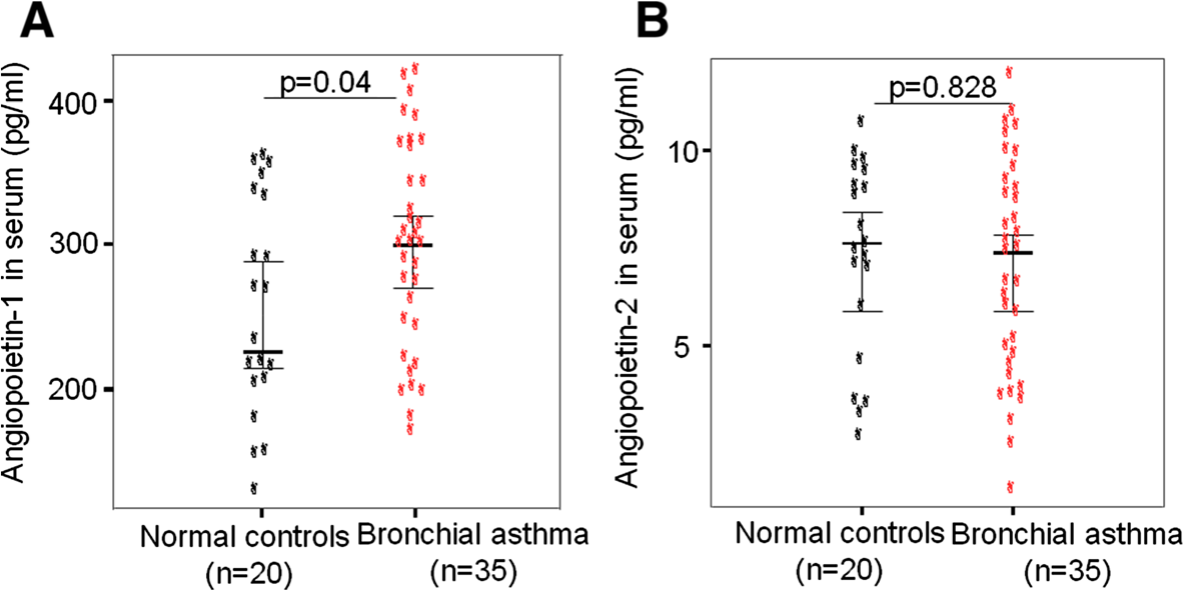
Serum angiopoietin 1 (A) and angiopoietin 2 (B) levels in patients with asthma and healthy controls.

**Figure 2:**
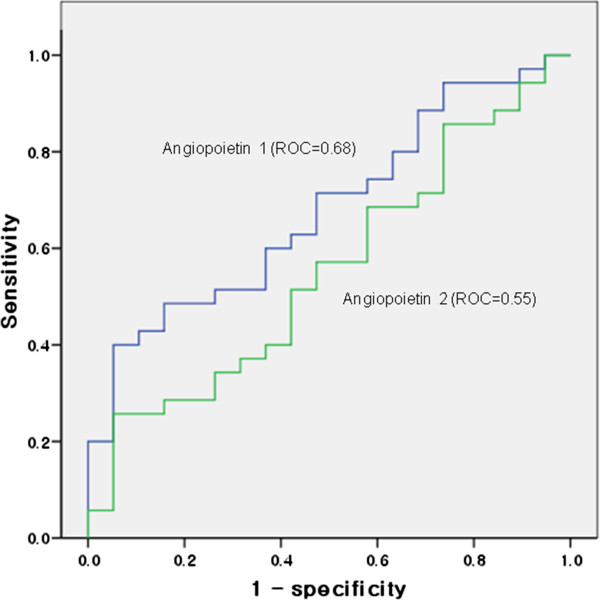
**Serum angiopoietin 1 and angiopoietin 2 receiver operating characteristic curves.** Those plot points in the left uppermost region represent more accurate values.

The serum Ang-1 levels were significantly elevated in the patients with asthma compared to those in the control subjects (293.9 ± 13.8 pg/mL vs. 248.3 ± 16.2 pg/mL, respectively, p = 0.04; Figure 1). The serum Ang-2 levels did not differ between the two groups (7.35 ± 0.57 pg/mL vs. 7.31 ± 0.80 pg/mL, respectively; Figure 1). The AUC for serum angiopoietins indicated that the serum Ang-1 levels (0.68) were more sensitive and specific than the serum Ang-2 levels (0.55) for differentiating patients with asthma from control subjects (Figure 2).

**Figure 3:**
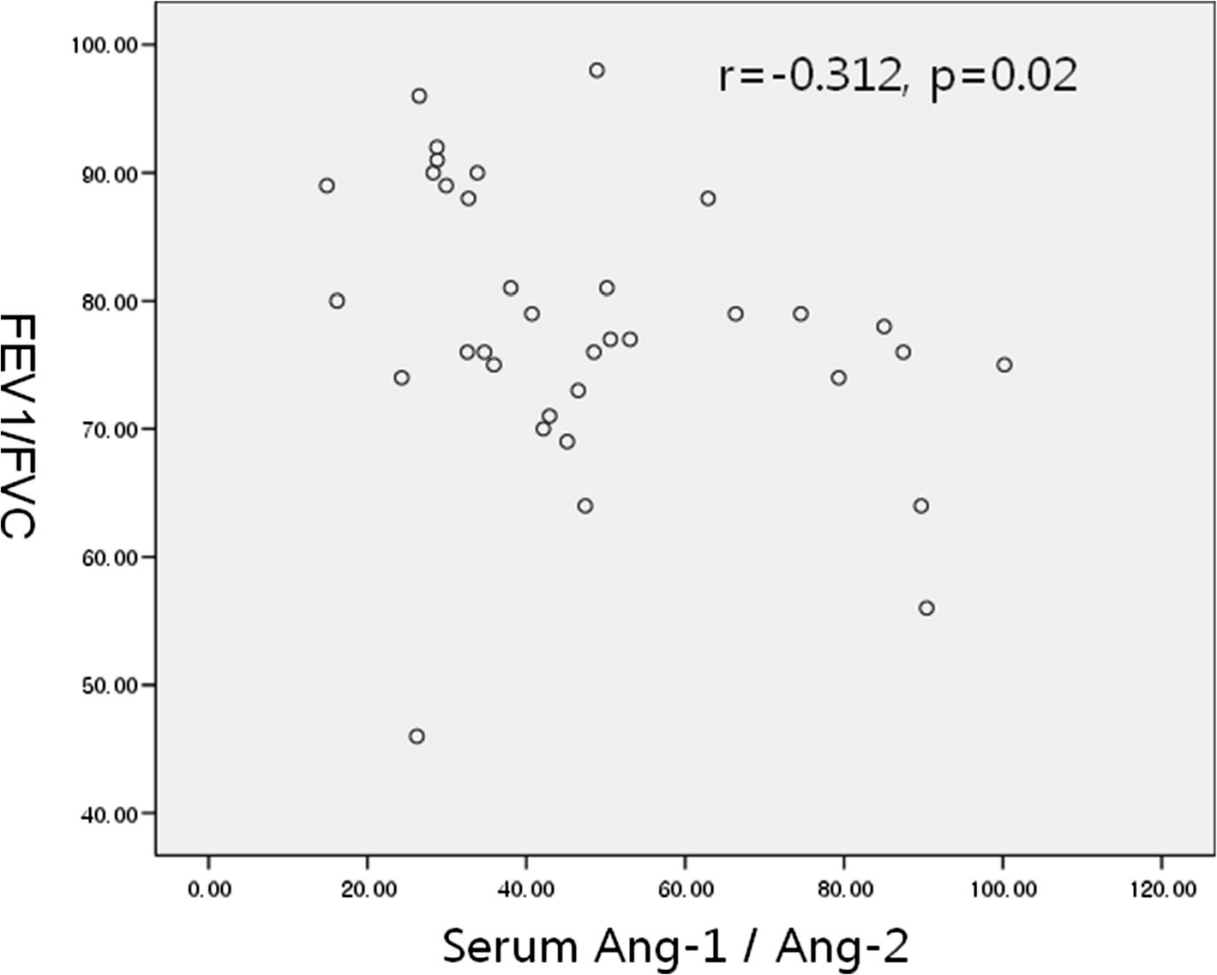
Association of the angiopoietin 1/angiopoietin 2 ratio with FEV1/FVC in patients with asthma.

**Figure 4:**
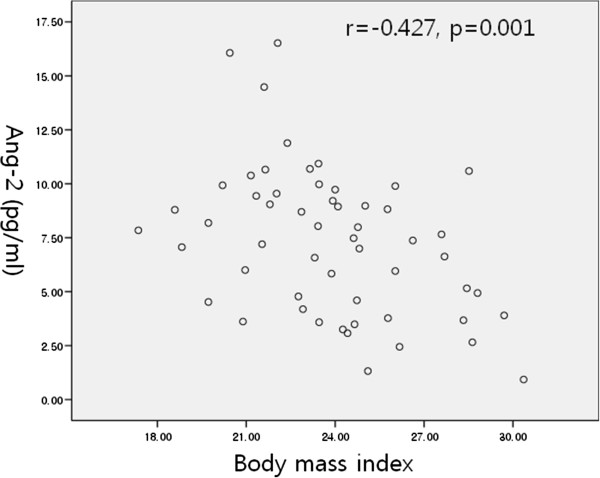
Relationship between angiopoietin 2 and body mass index in patients with asthma.

There were no differences between the two groups in terms of atopy, IgE level, smoking status, or sex. The FEV1, FVC, FEV1/FVC, and PC20 methacholine were lower in patients with asthma compared to control subjects. The serum Ang-1/Ang-2 ratio was correlated with the FEV1/FVC ratio (all subjects: r = -0.298, p = 0.027; asthmatic patients: r = -0.312, p = 0.02; Figure 3). Serum Ang-2 was correlated with BMI (all subjects: r = -0.427, p = 0.001; asthmatic patients: r = -0.353, p = 0.038; Figure 4). Serum Ang-1 and Ang-2 levels were not correlated with clinical parameters such as atopy, IgE level, and sputum eosinophilia.

## Discussion

Our results indicate that the serum Ang-1 levels were higher in asthmatics than in healthy subjects, and that the serum Ang-1/Ang-2 ratio was correlated with lung function, suggesting that angiopoietins are useful markers for the diagnosis of asthma.

Angiogenesis and vascular remodeling are key events in fundamental physiological processes, including growth and development, wound healing, organ regeneration, inflammation, and reproduction [29-31]. Increased size and numbers of bronchial blood vessels are also present, suggesting that angiogenesis is an essential component of tissue growth and remodeling in asthma [32]. Blood vessels are crucial for embryonic organ growth and the repair of wounded tissues in adults because they carry oxygen to the newly formed tissue. Angiogenesis is an important event in the development of allergic inflammation, as well as in the pathophysiology of tissue remodeling in asthma [6].

Angiogenesis is tightly regulated by a number of factors [28,33]. Ang-1 is predominantly expressed in perivascular cells during development but is constitutively expressed in adult tissues [34]. The role of Ang-2, the second Tie2 binding partner, appears to be more complex. In contrast to Ang-1, Ang-2 is only minimally expressed (or not expressed at all) in most normal adult tissues. However, it is strongly and rapidly upregulated in endothelial cells at sites of intensive vessel remodeling, including the female reproductive system and tumors [35].

The bronchial submucosa of patients with asthma has increased numbers of vessels in large as well as medium and small airways, which occupy a larger percentage of area than in normal subjects [5,36,37]. Airway vascular remodeling and inflammation may be responsible for increased bronchial blood flow [38] and exhaled breath temperature gradients in patients with asthma [39].

Ang-1 inhibits vascular permeability without affecting vascular morphology. Endothelial cells need to loosen the interendothelial cell contacts to emigrate from their resident sites [40]. Ang-2, an inhibitor of Tie2 signaling, may be involved in detaching smooth muscle cells and loosening the matrix [40]. Once the intracellular contacts have been loosened, VEGF initiates endothelial network organization and Ang-1 stabilizes the newly formed vessels [40].

In patients, circulating Ang-2, VEGF, and von Willebrand factor (VWF) levels are increased during ALI/ARDS or sepsis [11,13,41]. High Ang-2 levels correlate with impaired pulmonary gas exchange [11]. Circulating Ang-2 is associated with pulmonary permeability edema, and the occurrence and severity of ALI/ARDS in patients with and without sepsis [42]. The correlation of Ang-2 with VWF suggests the activated endothelium as a common source [42].

In our patients, the levels of circulating serum Ang-2 did not differ between normal controls and asthmatic patients, whereas the serum Ang-1 levels were increased in asthmatic patients. These results support the hypothesis that high Ang-2 levels, which antagonize the protective role of Ang-1, are involved in the increased pulmonary permeability that leads to asthma [11,13,41]. The serum Ang-1/Ang-2 ratio was related to the severity of lung impairment, suggesting a contributory role for Ang-1/Ang-2 in the pathogenesis of bronchial asthma. However, additional studies, including serial serum and alveolar compartment measurements of Ang-1/Ang-2 and blocking studies, are necessary to confirm this hypothesis. The serum Ang-2 levels in our patients with asthma are comparable to those reported in patients with severe asthma by Tseliou et al. [20], who reported higher Ang-1 and Ang-2 levels in patients with severe asthma. This discrepancy may be explained by the patient recruitment and serum sampling protocols used in our study. In animal models, Ang-1 levels were shown to be decreased in asthma and to protect against airway inflammation and hyperreactivity in asthma [43]. In contrast to the study of Simon [43], the serum Ang-1 levels in the present study were increased in patients with asthma compared to the controls, suggesting a difference in angiopoietin-producing cells between humans and other animals.

Interestingly, we found that serum Ang-2 was negatively correlated with BMI, suggesting that obesity reduces the serum levels of Ang-2 produced by endothelial cells and monocytes, resulting in imbalanced angiopoietin function. This study has a number of limitations related to the small number of patients and the retrospective nature of the study. Therefore, additional studies are needed of larger cohorts of asthma patients with different disease severities.

## Conclusions

The serum Ang-1 levels were higher in the asthmatic subjects than in the controls, and the serum Ang-1/Ang-2 ratio was correlated with lung function, indicating that serum angiopoietins may aid in the diagnosis of asthma.

## Competing interests

The authors declare that they have no competing interests.

## Authors’ contributions

MKY carried out the immunoassays, drafted the manuscript. LPH performed the statistical analysis. PSW and PCS participated in the acquisition of clinical data and in its design of the study. JAS conceived of the study, participated in its design and coordination and helped to draft the manuscript. All authors read and approved the final manuscript.

## Pre-publication history

The pre-publication history for this paper can be accessed here:

http://www.biomedcentral.com/1471-2466/14/143/prepub
